# Slow-Time Code Design for Space-Time Adaptive Processing in Airborne Radar

**DOI:** 10.3390/e23091169

**Published:** 2021-09-05

**Authors:** Shiyi Li, Na Wang, Jindong Zhang, Chenyan Xue, Daiyin Zhu

**Affiliations:** 1 Key Laboratory of Radar Imaging and Microwave Photonics, Ministry of Education, Nanjing University of Aeronautics and Astronautics, Nanjing 210016, China; wang_na@nuaa.edu.cn (N.W.); zhangjd@nuaa.edu.cn (J.Z.); xuechenyan@nuaa.edu.cn (C.X.); zhudy@nuaa.edu.cn (D.Z.); 2 Leihua Electronic Technology Research Institute, Aviation Industry Corporation of China, Wuxi 214063, China

**Keywords:** code design, space-time adaptive processing, moving target detecting, optimization algorithm

## Abstract

Space-time adaptive processing (STAP) techniques have been motivated as a key enabling technology for advanced airborne radar applications. In this paper, a slow-time code design is considered for the STAP technique in airborne radar, and the principle for improving signal-to-clutter and noise ratio (SCNR) based on slow-time coding is given. We present two algorithms for the optimization of transmitted codes under the energy constraint on a predefined area of spatial-frequency and Doppler-frequency plane. The proposed algorithms are constructed based on convex optimization (CVX) and alternating direction (AD), respectively. Several criteria regarding parameter selection are also given for the optimization process. Numerical examples show the feasibility and effectiveness of the proposed methods.

## 1. Introduction

Traditional space-time adaptive processing (STAP) involves multi-dimensional adaptive filtering which combines signals from several antenna elements and from multiple pulse repetitions to suppress clutter, interference, and noise in both space and time. It is well known that STAP improves detection performance of targets in both main lobe and side lobe clutter and in jamming interference environments. A great deal of attention has been given to STAP algorithms and much of the work has been done in the past three decades [1,2].

The signal design for radar performance improvement has been an active area of research in recent decades. However, the majority of previous works have considered pulse compression radar and clutter-free scenarios. For detecting a particular target in the presence of additive signal-dependent noise, waveform optimization theory, developed by Guerci [3,4,5], is evaluated in terms of the signal-to-interference-plus-noise ratio (SINR) under a particular model of the system, interference, clutter, and targets. The suboptimal solution of the phase-coded waveform for detecting a particular target in additive noise was proposed in [6]. For designing code for multiple-input multiple-output (MIMO) radar, [7] proposed average and worst-case performance metrics and several algorithms to solve highly nonconvex design problems.

It should be pointed out that waveform design problem for STAP has been discussed from the viewpoint of fast-time coding. In [8,9], waveform design and waveform scheduling problems in STAP for airborne radar are formulated with a cost function, and least-squared solutions for the designed waveform were obtained. In literature [10], the joint design of receive filter and waveform for the waveform adaptive radar STAP problem was solved by an alternating minimization algorithm. The STAP waveform design technique has also been applied to MIMO radar. Literature [11] improved the worst-case detection performance of STAP processing with a new diagonal loading (DL) based iterative approach. In [12,13], joint design problems of transmitting waveforms and the receiving filter were studied in terms of SCNR maximization and a manifold-based alternating optimization (MAO) method and an iterative algorithm based on the minorize-maximization (MM) technique were proposed respectively.

In this paper, slow-time coding for STAP for improving SCNR of STAP output is discussed. The rest of this work is organized as follows. Section 2 discusses the STAP model and derives the STAP filter and SCNR. Section 3 proposes two optimization methods for optimizing the output SCNR on average metric, i.e., CVX and AD. Section 4 demonstrates the proposed algorithms by extensive simulation experiments. Finally, concluding remarks and directions for future research are presented in Section 5.

In the following paragraphs the boldface upper case letters denote matrices; boldface lower-case letters denote column vectors, and italics denote scalars. ℂ denotes the complex field. The superscripts $\;{\left(.\right)}^{T}$, $\;{\left(.\right)}^{*}$, and ${\left(.\right)}^{H}$ denote transpose, complex conjugate, and conjugate transpose, respectively. The symbol ⊙ stands for the Hadamard (element-wise) product of matrices and ⊗ denotes the Kronecker product of matrices. tr(.) is the trace of a square matrix argument. $\mathrm{Diag}\left(.\right)$ denotes forming a diagonal matrix whose diagonal entries are formed by a vector, whereas diag(·) denotes the vector formed by collecting the diagonal entries of the matrix, E{·} stands for the statistical expectation operator, ≽ is positive semidefinite, ≻ is positive-definite, and ${\left\|.\right\|}_{F}$ indicates the Frobenius norm.

## 2. STAP Model

**Figure 1:**
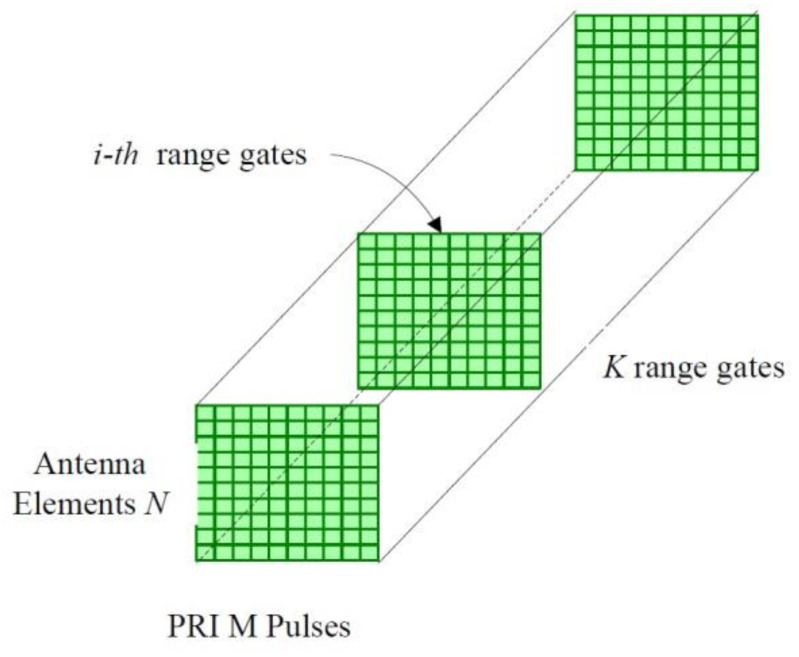
Phased-array radar CPI data cube.

The system under consideration is a pulsed Doppler radar residing on an airborne platform. The radar antenna is a uniformly spaced linear array antenna consisting of *N* elements. Radar returns are collected in a coherent processing interval (CPI), which is referred to as the 3-D radar data cube shown in Figure 1, where K denotes the number of samples collected to cover the range interval. The data are then processed at one range of interest, which corresponds to a slice of the CPI data cube. This slice is a M×N matrix which consists of N×1 spatial snapshots for Mpulses at the range of interest. It is convenient to stack the matrix column-wise to form the NM×1 vector $r_{i}$, termed the i th range gate space-time snapshot, 1≤i≤K.

The radar space-time snapshot is then expressed for each of the two hypotheses in the following form:

(1) $$\left\{\begin{array}{c} H_{0}\;:\;r_{i}=c_{i}+n_{i}\\ H_{1}\;:\;r_{i}=\alpha s+c_{i}+n_{i} \end{array}\right.$$

where α is a zero-mean complex Gaussian random variable with variance $\sigma_{s}^{2}$, s denotes the echo signal, and $c_{i}$ and $n_{i}$ are clutter and noise, respectively. These three components are assumed to be mutually uncorrelated.

The vector s is the NM×1 normalized space-time steering vector in the space-time look-direction and defined as [1] (p. 16):

(2) $$s=b_{t}\;⊗\;a_{t}$$

where $b_{t}$ is the corresponding M-dimensional temporal steering vector given by:

(3) $$b_{t}=[1\;,e^{j2\pi f_{t}},\;\ldots \;,e^{j2\pi (M-1)f_{t}}{\;]}^{T}$$

and $a_{t}$ is the N-dimensional spatial steering vector and given by:

(4) $$a_{t}=[1\;,e^{j2\pi \theta_{t}},\;\ldots \;,e^{j2\pi (N-1)\theta_{t}}{\;]}^{T}$$

We define $f_{t}=\omega_{t}T_{r}$ as normalized Doppler frequency and $\theta_{t}=d\cos\varphi \sin\phi /\lambda$ as special frequency, where $\omega_{t}$ is target Doppler frequency, $T_{r}$ is pulse repetition interval, d is interelement spacing, φ is elevation angle, ϕ is azimuth angle, λ is radar operating wavelength.

The clutter $c_{i\;}$ can be represented as

(5) $$c_{i\;}=\sum_{l=1}^{N_{c}}\xi_{l}^{c}b(f_{l}^{c})⊗a(\theta_{l}^{c})$$

where $N_{c}$ denotes the number of the clutter patches, $\xi_{l}^{c}$ is the power of reflected signal by the lth clutter patch. $a(\theta_{l}^{c})$ and $b(f_{l}^{c})$, respectively, denote the spatial steering vector with the spatial frequency $\theta_{l}^{c}$ and the temporal steering vector with the normalized Doppler frequency $f_{l}^{c}$ for the lth clutter patch. Thus, the clutter covariance matrix can be expressed as

(6) $$C=E\{c_{i}c_{i}^{H}\}=\sum_{l=1}^{N_{c}}{\left(\xi_{l}^{c}\right)}^{2}[b(f_{l}^{c})b{\left(f_{l}^{c}\right)}^{H}]⊗[a(\theta_{l}^{c})a{\left(\theta_{l}^{c}\right)}^{H}]$$

The noise covariance matrix $M=\;E\left\{n_{i}n_{i}^{H}\;\right\}$ can be written as a scaled identity matrix $\sigma_{n}^{2}I_{NM}$ where $\sigma_{n}^{2}$ is the noise power.

To detect the presence of targets, each range bin is processed by an adaptive 2D beam former (to achieve maximum output SINR) followed by a hypothesis test to determine the target presence or absence.

The optimum full-rank STAP filter obtained by an unconstrained optimization of the SCNR is given as follows [1] (p. 57):

(7) $$w_{\mathrm{opt}}=\kappa {\left(C+M\right)}^{-1}s$$

where κ is a constant number. The corresponding optimum SCNR is given by [1] (p. 62):

(8) $$\mathrm{SCNR}=s^{H}{\left(C+M\right)}^{-1}s$$

## 3. Slow-Time Code Design for STAP Radar

### 3.1. Problem Formulation

In this section, the radar system is considered to transmit a series of weighted pulse. Using Equation (1), the target detection problem can also be cast as the following binary hypothesis test

(9) $$\left\{\begin{array}{c} H_{0}\;:\;r_{i}=c_{i}⊙p+n_{i}\\ H_{1}\;:\;r_{i}=\alpha s\;⊙p+c_{i}⊙p+n_{i} \end{array}\right.$$

where p=a⊗1, $a{=[a}_{0}{\;a}_{1}\;\ldots {\;a}_{M-1}{]}^{T}$ are the transmit weights that are to be optimally designed, and $1={\left[1\;1\;\ldots \;1\right]}^{T}$. The performance of the optimum detector depends on the following SCNR

(10) $$\mathrm{SCNR}=(s⊙p{)}^{H}{\left(PCP^{H}+M\right)}^{-1}(s\;⊙p)$$

where $P=\mathrm{Diag}\left(p\right)$.

Slow-time code design to improve the detection performance of the STAP system for a known target Doppler frequency shift $f_{t}$ and arrival angle $\theta_{t}$ can be accomplished by maximizing the following performance metric:

(11) $$\begin{array}{ll} \mathrm{SCNR} & =({s⊙p}^{H})({\mathrm{PCP}}^{H}+{+M)}^{-1}(s\;⊙p)\\ & =\mathrm{tr}(P^{H}{(\mathrm{PCP}}^{H}{+M)}^{-1}{\mathrm{Pss}}^{H})\\ & =\mathrm{tr}\{((P^{H}MP)-1{+C)}^{-1}{\mathrm{ss}}^{H}\} \end{array}$$

It can be noted that a phase-shift version of the code vector p will not alter the SCNR value and the detection performance will also not be improved.

In cases where the Doppler frequency shift and direction of arrival (DOA) of the interested target lie in a certain area, we consider the following design metric:

(12) $$\mathrm{tr}\{{({(P^{H}MP)}^{-1}+C)}^{-1}S\}$$

where $S=E\{ss^{H}\}$. To optimize the detection performance for a STAP radar, the metric above can be maximized under an energy constraint:

(13) $$\begin{array}{l} \underset{P}{\max}\;\mathrm{tr}\{{({(P^{H}MP)}^{-1}+C)}^{-1}S\}\\ s.t.\;\mathrm{tr}(PP^{H})=e \end{array}$$

where e denotes the transmission energy.

### 3.2. Algorithm Based on Convex Optimization

Because of the objective function in problem (13) is highly nonconvex, this problem is difficult to tackle. In this section, the CVX algorithm is proposed based on semi definite programming (SDP) relaxation.

In order to reformulate this nonconvex problem as a convex problem, we introduce the auxiliary variable $X≜P^{H}M^{-1}P$. We also set $S=VV^{H}=\sum_{k=1}^{\delta }v_{k}v_{k}^{H}$ and $C=TT^{H}$ where δ=rank(S), $V=[v_{1}\;v_{2}\ldots v_{\delta }]\in {\mathbb{C}}^{NM\times \delta }$,$T\in {\mathbb{C}}^{NM\times NM}$. Using the matrix inversion lemma in [14], we have:

(14) $$\mathrm{tr}({\left(X^{-1}+C\right)}^{-1}S)=\sum_{k=1}^{\delta }\{v_{k}^{H}Xv_{k}-v_{k}^{H}XT{\left(I+T^{H}XT\right)}^{-1}T^{H}Xv_{k}\}$$

Using auxiliary variables $b_{k=1}^{\delta }$ and Schur complement theorem [15], the maximization of Equation (14) can be achieved by minimization of $\sum_{k=1}^{\delta }b_{k}$ with a constraint as follows:

(15) $$b_{k}\geq -v_{k}^{H}Xv_{k}+v_{k}^{H}XT{\left(I+T^{H}XT\right)}^{-1}T^{H}Xv_{k}\Leftrightarrow \left[\begin{array}{cc} b_{k}+v_{k}^{H}Xv_{k} & v_{k}^{H}XT\\ T^{H}Xv_{k} & I+T^{H}XT \end{array}\right]⪰0$$

Note that the energy constraint in problem Equation (13) with respect to X is $\mathrm{tr}(X{\left(M^{-1}⊙I\right)}^{-1})\leq e$ and observe that X≻0. Then, the nonconvex problem of Equation (13) can be reformulated as a semi definite programming problem in a relaxed form:

(16) $$\begin{array}{ll} \underset{X,\{b_{k=1}^{\delta }\}}{\min}\;\; & \sum_{k=1}^{\delta }b_{k}\\ s.t.\;\; & \left[\begin{array}{cc} b_{k}+v_{k}^{H}Xv_{k} & v_{k}^{H}XT\\ T^{H}Xv_{k} & I+T^{H}XT \end{array}\right]⪰0,\forall k\\ & X≻0,\mathrm{tr}(X{\left(M^{-1}⊙I\right)}^{-1})\leq e \end{array}$$

The above problem can be solved by CVX package in polynomial time. 

Next, we use X to approach a by introducing the auxiliary unitary matrix Q, and this leads to the minimization problem:

(17) $$\begin{array}{ll} \underset{P,Q}{\min}\;||X^{1/2}Q-P^{H}M^{-1/2}|{|}_{F}^{2}\Leftrightarrow \underset{P,Q}{\min} & \mathrm{tr}(X)+\mathrm{tr}(P^{H}M^{-1}P)-2ℜ(\mathrm{tr}(M^{-1/2}PX^{1/2}Q))\\ s.t.\;QQ^{H}=I\;\;\;\;\;\;\;s.t. & QQ^{H}=I \end{array}$$

Considering the two variables in the optimization problem in Equation (17), the alternating direction method is considered for use here. Therefore, we can optimize one variable when the other is fixed, and Equation (17) can be split into two optimization problems:

(18) $$\begin{array}{l} P1:\underset{Q}{\max}\;ℜ(\mathrm{tr}(M^{-1/2}PX^{1/2}Q))\\ s.t.\;QQ^{H}=I \end{array}$$

and

(19) $$P2:\underset{p}{\min}\;p^{H}{\left(M^{-1}⊙I\right)}^{-1}p-2ℜ(d^{H}p)$$

where $d=\mathrm{diag}(X^{1/2}Q^{*}M^{-1/2})$.

Problem 1 is an orthogonal Procrustes problem [16], let $V_{1}\Sigma V_{2}^{H}$ represent the singular value decomposition (SVD) of $M^{-1/2}PX^{1/2}$, and set unitary matrix $Z=V_{2}^{H}QV_{1}$ and $\Sigma =\mathrm{Diag}({\left[\sigma_{1}\;\sigma_{2}\;\ldots \;\sigma_{N}\right]}^{T})$, then we have:

(20) $$\begin{array}{l} ℜ(\mathrm{tr}(M^{-1/2}PX^{1/2}Q))=ℜ(\mathrm{tr}(V_{1}\Sigma V_{2}^{H}Q))=ℜ(\mathrm{tr}(\Sigma V_{2}^{H}QV_{1}))\\ =ℜ(\mathrm{tr}(\Sigma Z))=\sum_{i=1}^{N}ℜ(z_{ii})\sigma_{i}\leq \sum_{i=1}^{N}\sigma_{i} \end{array}$$

The above inequality becomes an equality if and only if $Z=I_{MN}$. Then, the solution of P1 is given by $Q=V_{2}V_{1}^{H}$.

Problem 2 is an unconstrained quadratic program. Note that the transmit code a is the optimized variable, and let $H=I_{M}⊗1_{N}$, then we have p=Ha.

Consequently, the P2 problem can also be given by:

(21) $$P2\prime :\underset{a}{\min}\;a^{H}H^{H}{\left(M^{-1}⊙I\right)}^{-1}Ha-2ℜ(d^{H}Ha)$$

As a result, the solution is directly given by $a={\left(H^{H}(M^{-1}⊙I)H\right)}^{-1}H^{H}d$.

The whole optimization process is summarized as follows in Table 1:

### 3.3. Algorithm Based on Alternating Direction Method

In this section, the AD algorithm is given based on the auxiliary variable method and alternating direction method.

It follows from Section 3.1 that $S=VV^{H}$. As a result, the objective function in problem Equation (13) can be converted to:

(22) $$\begin{array}{l} \;\mathrm{tr}\{{\left(({P^{H}MP)}^{-1}+C\right)}^{-1}S\}\\ =\mathrm{tr}\{V^{H}P^{H}{(\mathrm{PCP}}^{H}{+M)}^{-1}PV\} \end{array}$$

Let $\Theta =\theta I-V^{H}P^{H}{\left(PCP^{H}+M\right)}^{-1}PV≽0$. The choice of the parameter θ will be described later. As a consequence, the maximization problem in Equation (13) can be transformed to

(23) $$\begin{array}{l} \underset{P}{\min}\;\mathrm{tr}\{\Theta \}\\ s.t.\;\mathrm{tr}(PP^{H})=e \end{array}$$

For solving this problem, we start with the following problem with respect to the auxiliary variable Y:

(24) $$\begin{array}{ll} \underset{Y\;P}{\min} & \mathrm{tr}\{\Xi \}\\ s.t. & Y^{H}U=I_{\delta \times \delta }\;\\ & \mathrm{tr}(PP^{H})=e \end{array}$$

where $\Xi =Y^{H}RY$, $U=[I_{\delta \times \delta };\;0_{NM\times \delta }{]}^{T}$ and $R=\left[\begin{array}{cc} \theta I_{\delta \times \delta } & V^{H}P^{H}\\ PV & PCP^{H}+M \end{array}\right]$.

Considering the two variables in the optimization problem in (24), alternating direction method is considered for use here. It can be proven that for fixed P the minimizer of the above problem is given by:

(25) $$Y_{0}=R^{-1}U{\left(U^{H}R^{-1}U\right)}^{-1}$$

Note that ${\left(U^{H}R^{-1}U\right)}^{-1}$ exists. Then, let $Y_{0}=R^{-1}U{\left(U^{H}R^{-1}U\right)}^{-1}$, and note that $Y_{0}^{H}U=I_{\delta \times \delta }$. Let $Y=Y_{0}+\Delta$, where $\Delta \in {\mathbb{C}}^{(NM+\delta )\times \delta }$ satisfies $\Delta^{H}U=0$, so that Y also satisfies the constraint. Then,

(26) $$Y^{H}RY=Y_{0}^{H}RY_{0}+Y_{0}^{H}R\Delta +\Delta^{H}RY_{0}+\Delta^{H}R\Delta$$

where the two middle terms are equal to zero:

(27) $$\Delta^{H}RY_{0}=\Delta^{H}RR^{-1}U{\left(U^{H}R^{-1}U\right)}^{-1}=0$$

Hence, we have

(28) $$Y^{H}RY-Y_{0}^{H}RY_{0}=\Delta^{H}R\Delta \geq 0$$

as R is positive definite. It follows from (28) that the optimal solution of this problem is given by Equation (25).

According to the block matrix inverse formula in [17], we have:

(29) $$\Theta^{-1}=U^{H}R^{-1}U$$

It follows from Equation (29) that $Y^{H}RY=\Theta$, when $Y=Y_{0}$. Consequently, for fixed $Y=Y_{0}$, the minimization of tr{Ξ} in problem Equation (24) with respect to P leads to a decrease of tr{Θ}. Thus, problem Equation (23) can be solved by a cyclic iterative method. In each loop, Y is set to $Y_{0}$ firstly, and secondly, minimizer P is obtained by solving problem Equation (24) for fixed Y.

Let $Y={\left[Y_{1\;\delta \times \delta };Y_{2\;NM\times \delta }\right]}^{T}$, we can also have

(30) $$\begin{array}{ll} Y^{H}RY & =[Y_{1}^{H}\;Y_{2}^{H}]\left[\begin{array}{cc} \theta I_{\delta \times \delta } & V^{H}P^{H}\\ PV & PCP^{H}+M \end{array}\right]\left[\begin{array}{c} Y_{1}\\ Y_{2} \end{array}\right]\\ & =\theta Y_{1}^{H}Y_{1}+Y_{2}^{H}PVY_{1}+Y_{1}^{H}V^{H}P^{H}Y_{2}+Y_{2}^{H}(PCP^{H}+M)Y_{2} \end{array}$$

and

(31) $$\mathrm{tr}(Y^{H}RY)=\mathrm{tr}(\theta Y_{1}^{H}Y_{1}+Y_{2}^{H}MY_{2})+p^{H}(C^{T}⊙(Y_{2}Y_{2}^{H}))p+2ℜ(g^{H}p)$$

where $g=\mathrm{diag}(V^{*}Y_{1}^{*}Y_{2}^{T})$. It can be seen problem Equation (24) with respect to P is a convex quadratically constrained quadratic program. Note that p=Ha and $H=I_{M}⊗1_{N}$. Then, above problem can also be given by:

(32) $$\begin{array}{l} \underset{a}{\min\;}a^{H}H^{H}(C^{T}⊙(Y_{2}Y_{2}^{H}))Ha+2ℜ(g^{H}Ha)\;\\ \;s.t.\;a^{H}a=\frac{e}{N} \end{array}$$

This problem can be tackled by Lagrange multiplier method. Let

(33) $$L\left(a,\mu \right)=a^{H}H^{H}(C^{T}⊙(Y_{2}Y_{2}^{H}))Ha+2ℜ(g^{H}Ha)+\mu (a^{H}a-\frac{e}{N})$$

be Lagrange function with non-negative multiplier μ. For fixed μ, the unconstrained minimizer a is:

(34) $$a_{\mu }=-{\left(H^{H}(C^{T}⊙(Y_{2}Y_{2}^{H}))H+\mu I\right)}^{-1}H^{H}g$$

Substituting Equation (34) into Equation (33), we obtain a concave function with respect to μ:

(35) $$L\left(a_{\mu },\mu \right)=-gH^{H}{\left(H^{H}(C^{T}⊙(Y_{2}Y_{2}^{H}))H+\mu I\right)}^{-1}H^{H}g-\mu \frac{e}{N}$$

Then, we can obtain the maximizer of μ by solving the following equation:

(36) $$gH^{H}{\left(H^{H}(C^{T}⊙(Y_{2}Y_{2}^{H}))H+\mu I\right)}^{-2}H^{H}g=\frac{e}{N}$$

by using the Newton method [18].

In addition, the parameter θ should satisfy:

(37) $$\theta \geq \lambda_{\max}(V^{H}P^{H}{(\mathrm{PCP}}^{H}{+M)}^{-1}PV)$$

Note that

(38) $$\begin{array}{ll} \lambda_{\max}(V^{H}P^{H}({\mathrm{PCP}}^{H}+M)-1PV) & \leq \lambda_{\max}(({C+P^{-1}MP^{-H})}^{-1}).\lambda_{\max}(S)\\ & \leq \frac{1}{\lambda_{\min}(C+P^{-1}MP^{-H})}.\lambda_{\max}(S) \end{array}$$

Furthermore, there exists

(39) $$\begin{array}{ll} \lambda_{\min}(C+P^{-1}MP^{-H}) & \geq \lambda_{\min}(C)+\lambda_{\min}(P^{-1}MP^{-H})\\ & \geq \lambda_{\min}(C)+\frac{\lambda_{\min}(M)}{e} \end{array}$$

As a result, the parameter θ should satisfy

(40) $$\theta \geq \frac{e\lambda_{\max}(S)}{\lambda_{\min}(M)+e\lambda_{\min}(C)}$$

where $\lambda_{\max}(.)$ and $\lambda_{\min}(.)$ denote the largest and smallest eigenvalues, respectively.

The whole optimization process is summarized as follows in Table 2:

## 4. Numerical Examples

In this section, we assess the proposed optimization algorithm using simulated radar data. The parameters of the simulated radar platform are shown in Table 3. The thermal noise is modeled as a Gaussian white noise with unity power. The clutter and target powers can be referred to the white noise power. For all simulations, the clutter-to noise-ratio (CNR) is fixed at 20 dB. All presented results are averages over 1000 independent Monte Carlo runs:

It must be pointed out that the optimization model in this paper can also be solved by heuristic algorithms, such as the genetic algorithm (GA). GA is easy to implement and it has been successfully applied to radar waveform design in the literature [19,20]. However, with increased variables in the optimization problem, and the effect of random initial values, slow convergence speed and unsatisfactory results may be obtained in practice. In the follow-up numeric experiments, the performance of the two proposed algorithms is compared with a GA approach, whose parameters are set as follows: initial population size S=40, crossover rate $P_{c}=0.95$, mutation rate $P_{m}=0.1$, and maximum number of genetic iterations is 150.

In the following numeric examples, both the average metric in Equation (12) and SCNR in Equation (11) are used to evaluate the optimization effect. Furthermore, to evaluate SCNR improvement by incorporating slow-time coding, the metric of ${\mathrm{IMP}}_{\mathrm{SCNR}}$ is defined as

(41) $${\mathrm{IMP}}_{\mathrm{SCNR}}=10\log_{10}\frac{{\mathrm{SCNR}}_{\mathrm{opt}}}{\mathrm{SCNR}}(dB)$$

where ${\mathrm{SCNR}}_{\mathrm{opt}}$ and SCNR denote calculated SCNR with optimized slow-time coding and without coding.

To optimize the slow-time code, the interested area in spatial-frequency and Doppler-frequency planes should be selected. We assume that the velocity vector is aligned with the array axis. Then, the clutter Doppler frequency is a liner function of spatial-frequency [1] (p. 25):

(42) $$f_{s}=2v_{a}T_{r}\theta_{s}/d$$

It follows from the parameters in Table 3 and Equation (42), ground clutter appears on a ridge of spatial-frequency and Doppler-frequency plane in STAP output where $f_{s}=\theta_{s}$. Thus, we pick a certain area which is parallel to the clutter ridge for optimizing the slow-time code. The relationship of the spatial-frequency $\theta_{s}$ and the normalized Doppler-frequency $f_{s}$ in the selected area is given by:

(43) fs=θ
s±Δ+[0:s−1]N

where Δ indicates the distance between clutter ridge and the interested area and s is the width of the interested area.

**Figure 2:**
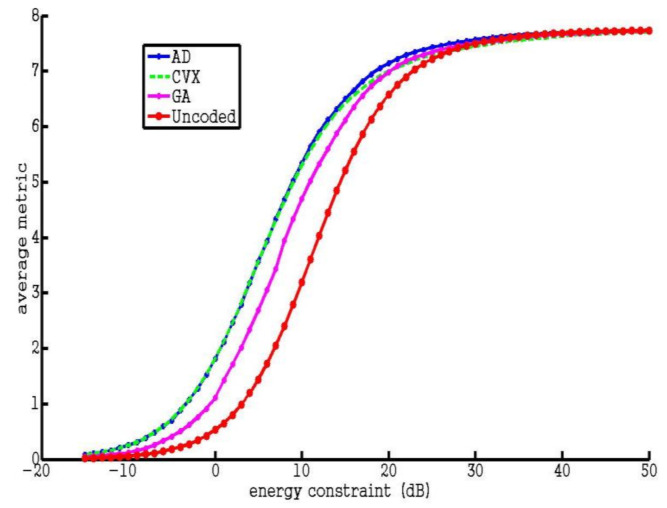
Average metric for 4 different codes vs. transmit energy.

Firstly, we compare the two proposed optimization algorithms and GA by studying the average metric of resultant codes under different energy constraints. Herein, we consider an example of code design for a certain area defined by Equation (43) with parameters Δ = 1 and s = 2. As can be seen from Figure 2, the average metrics of both proposed algorithms are significantly better than those of the uncoded system and GA. It also can be seen that the AD algorithm outperforms the CVX algorithm with slightly larger average metric, due to the optimality losses arising in the approximation process of CVX algorithm. We can also find the saturation phenomenon of the average metric when the energy constraint is sufficiently high.

**Figure 3:**
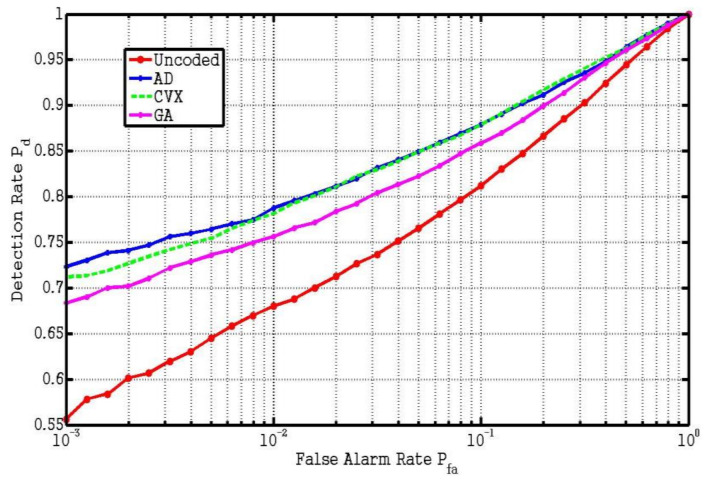
ROC of GLR detector for 4 different codes using Monte Carlo method.

Secondly, we use the generalized likelihood ratio (GLR) detector [21] to study the detection performance of the above 4 different codes. We assume that e=5, Δ = 1 and s = 2. The receiver operating characteristic (ROC) curve can be obtained by Monte Carlo method. First, 10,000 groups of random noise signals are generated to obtain the detection threshold η for each false alarm rate by Neyman–Pearson criterion. Then, the target signal is added to the 10,000 groups of noise signals to calculate the detection rate under each false alarm rate. As can be seen from the ROC curve in Figure 3, the target detection performance of two proposed resultant codes is much better than that of the uncoded system and GA, and only minor difference can be found between the two proposed algorithms.

**Figure 4:**
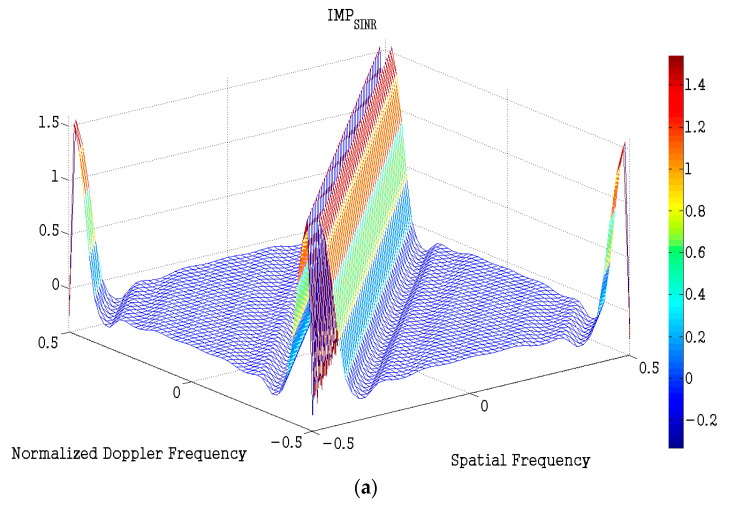

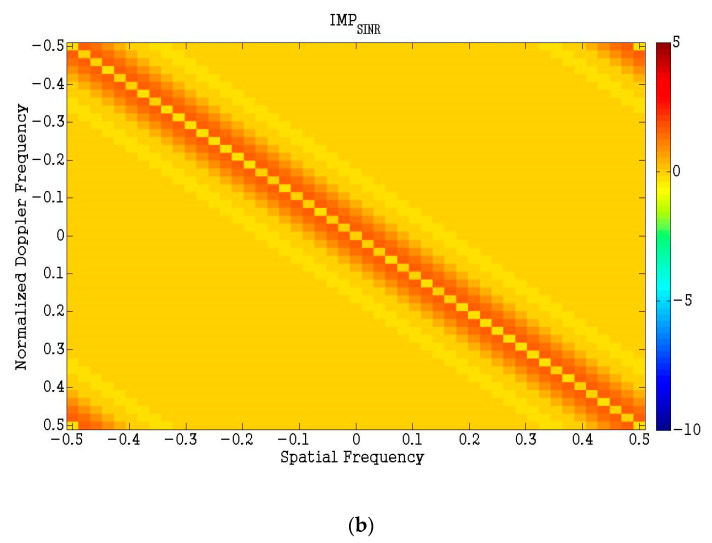
The metric of ${\mathrm{IMP}}_{\mathrm{SCNR}}$, (**a**) view from side look, (**b**) view from top look.

Thirdly, we use the resultant code generated by the AD algorithm to tackle the optimization effect at different spatial frequencies and Doppler frequencies. We assume that e=5, Δ = 1, and s = 2. Figure 4 shows the metric of ${\mathrm{IMP}}_{\mathrm{SCNR}}$ on the whole spatial-frequency and Doppler-frequency plane. About 2 dB SCNR improvement can be obviously seen form the area, which is parallel to the clutter ridge, and no SCNR loss appears on the clutter ridge. Therefore, slow-time coding can effectively improve slow moving target detection performance for the STAP radar system.

**Figure 5:**
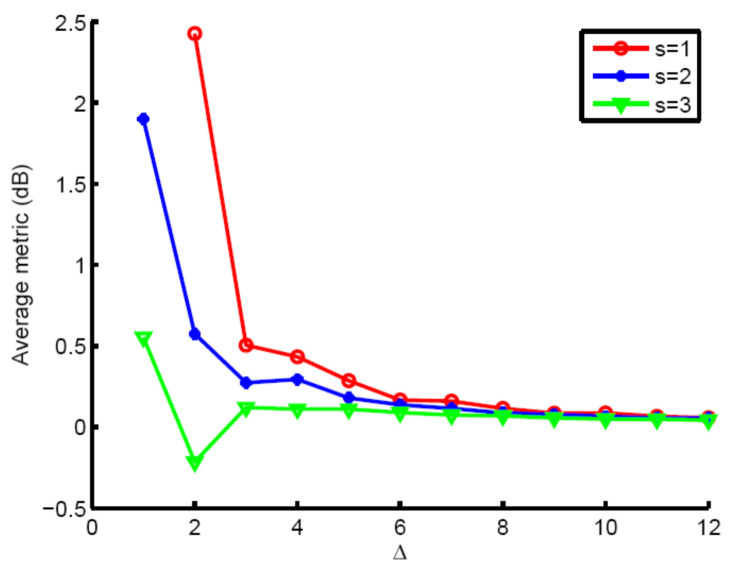
Average metric in dB scale vs. Δ.

Finally, we use the AD algorithm to study the influence of optimization area selection on the optimization effect through the next two experiments. We assume that e=5. Figure 5 demonstrates the average metric over the interested area. As Δ increases, the average metric decreases fast. Therefore, slow-time coding can only improve slow moving target detection. Meanwhile, s should be carefully chosen. s = 2 indicates a better performance.

**Figure 6:**
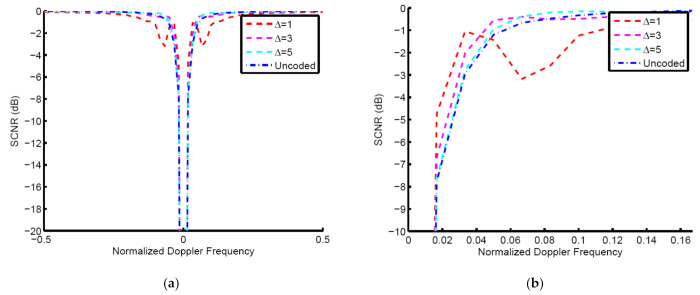
The result of SCNR vs. Doppler for side-looking case, (**a**) Doppler frequency interval of [−0.5, 0.5], (**b**) Doppler frequency interval of [0, 0.165].

Figure 6 shows the result of SCNR vs. Doppler for the side-looking case $\left(\theta {=0}^{o}\right)$. We assume that e=5 and s = 2. The resultant code of Δ = 1 has the highest SCNR in the normalized Doppler frequency interval of [0.02, 0.04]. The resultant codes of Δ = 3 and Δ = 5 present lower SCNR than the code of Δ = 1, but these values are still higher than the uncoded system result. This means that the selection of Δ depends on the target normalized Doppler frequency. When the target normalized Doppler frequency is less than 0.04, Δ = 1 can achieve better effect. Otherwise, Δ = 3 and Δ = 5 indicate a better performance.

## 5. Conclusions

The principle of STAP radar for improving SCNR based on slow-time coding is described in this paper. When slow-time code is optimized according to the selected area on spatial-frequency and Doppler frequency planes, the SCNR metric can be improved accordingly. Two optimization algorithms, i.e., CVX and AD, are proposed. Numerical examples show that the two proposed algorithms outperform GA, and the SCNR around the clutter ridge is improved significantly. Therefore, slow-time coding can optimize slow moving target detection performance.

## Figures and Tables

**Table 1:** The CVX algorithm for optimizing slow-time code.

Step 0:	Solve the SDP of Equation (16) to obtain X.
Step 1:	initialize $a^{\left(0\right)}$ using uniform code, k = 0;
Step 2:	solve P1 problem by $Q=V_{2}V_{1}^{H}$ and $V_{1}\Sigma V_{2}^{H}$ represent the singular value decomposition (SVD) of $M^{-1/2}PX^{1/2}$,
Step 3:	solve P2 problem by $a={\left(H^{H}(M^{-1}⊙I)H\right)}^{-1}H^{H}d$, and normalized a by $a^{\left(k\right)}=\sqrt{\frac{e}{Na^{H}a}}a$
Step 4:	set k = k + 1, repeat the steps 2∼3 until a certain stop criterion, e.g., $||a^{\left(k+1\right)}-a^{\left(k\right)}|{|}_{2}^{2}\leq \;\epsilon$, where ϵ is a predefined value.

**Table 2:** The AD algorithm for optimizing slow-time code.

Step 0:	initialize $a^{\left(0\right)}$ using uniform code, k = 0;
Step 1:	calculate the parameter θ by Equation (40);
Step 2:	calculate the matrix $R^{\left(k\right)}$;
Step 3:	set $Y^{\left(k\right)}=R^{(k)-1}U{\left(U^{H}R^{(k)\;-1}U\right)}^{-1}$ and calculate $g^{\left(k\right)}$
Step 4:	solve problem (32) by $a=-{\left(H^{H}(C^{T}⊙(Y_{2}Y_{2}^{H}))H+\mu I\right)}^{-1}H^{H}g$, calculate μ, and normalized a by $a^{\left(k\right)}=\sqrt{\frac{e}{Na^{H}a}}a$
Step 5:	set k = k + 1, repeat the steps 2∼4 until a certain stop criterion, e.g., $||a^{\left(k+1\right)}-a^{\left(k\right)}|{|}_{2}^{2}\leq \;\epsilon$, where ϵ is a predefined value.

**Table 3:** Airborne phased-array radar parameters.

Parameters	Value
Carrier frequency	10.0 GHz
System bandwidth	5 MHz
Pulse repetition frequency ($1/T_{r}$)	5000 Hz
Flight velocity ($v_{a}$)	37.5 m/s
Antenna array spacing (d)	1.5 cm
Elements of antenna array N	16
Number of pulses M	16
Clutter-to-noise ratio(CNR)	20 dB
Target mean power	20 dB

## Data Availability

The datasets generated during the current study are not publicly available but are available from the corresponding author on reasonable request.
